# 1,2-Bis[(3,5-diphenyl-1*H*-pyrazol-1-yl)meth­yl]benzene

**DOI:** 10.1107/S1600536812032801

**Published:** 2012-07-28

**Authors:** Lara C. Spencer, Ilia A. Guzei, Tebogo V. Segapelo, James Darkwa

**Affiliations:** a Department of Chemistry, University of Wisconsin-Madison, 1101 University Ave, Madison, WI 53706, USA; b Department of Chemistry, University of Johannesburg, Auckland Park Kingsway Campus, Johannesburg 2006, South Africa

## Abstract

The title compound, C_38_H_30_N_4_, a potentially mono- and bidentate ligand, does not seem to form palladium complexes similar to other poly(pyrazol-1-ylmeth­yl)benzenes due to the large steric size of the phenyl substituents on the pyrazole rings. The pyrazole rings have a 21.09 (5)° angle between their mean planes and exhibit a *trans*-like geometry in which the in-plane lone pairs of electrons on the 2-N nitrogen atoms point in opposite directions.

## Related literature

For information about poly(pyrazol-1-ylmeth­yl)benzenes and the metal complexes they form, see: Hartshorn & Steel (1995 ▶, 1997 ▶, 1998 ▶); Motsoane *et al.* (2007 ▶). For information on the related compounds 1,2-bis­[(3-(2,2′-bipyridin-6-yl)pyrazol-1-yl)meth­yl]benzene and 2,3-bis­[(3-(2-pyrid­yl)pyrazol-1-yl)meth­yl]naphthalene, see: Al-Rasbi *et al.* (2007 ▶); Paul *et al.* (2003 ▶). Geometrical parameters were checked with *Mogul* (Bruno *et al.*, 2002 ▶).
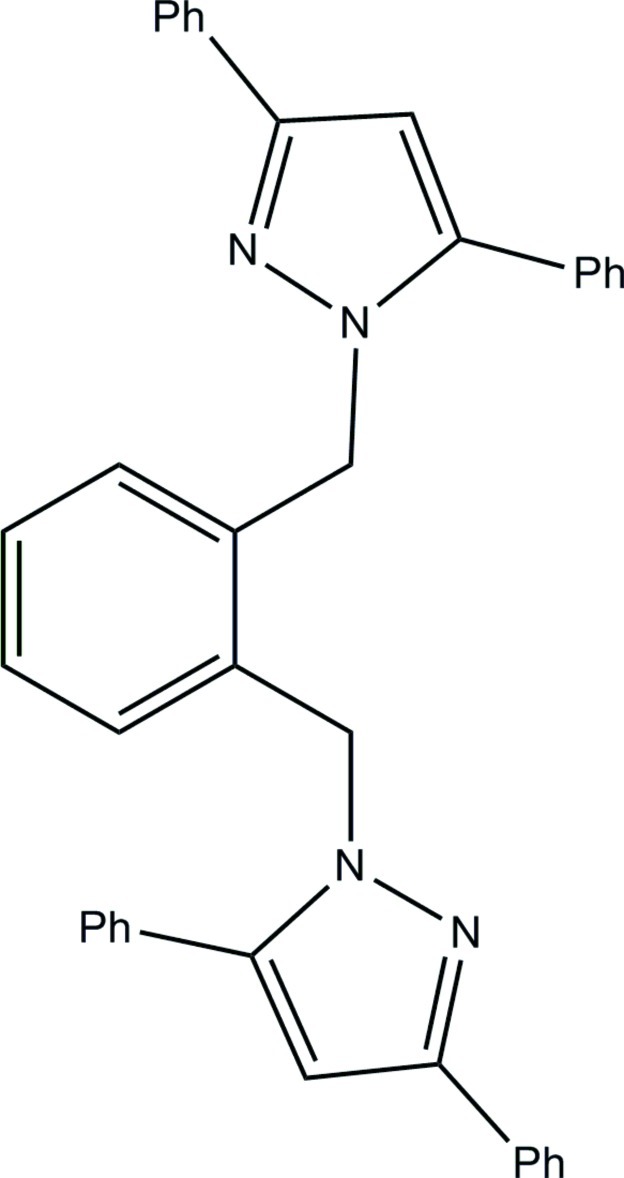


## Experimental

### 

#### Crystal data

C_38_H_30_N_4_
*M* *_r_* = 542.66
Monoclinic, 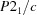

*a* = 14.5338 (2) Å
*b* = 13.6779 (2) Å
*c* = 15.0051 (2) Å
β = 110.102 (1)°
*V* = 2801.18 (7) Å^3^
*Z* = 4
Cu *K*α radiation
μ = 0.59 mm^−1^
*T* = 100 K
0.25 × 0.18 × 0.15 mm

#### Data collection

Bruker APEXII CCD diffractometer
Absorption correction: multi-scan (*SADABS*; Bruker, 2007 ▶) *T*_min_ = 0.891, *T*_max_ = 0.943
44869 measured reflections
5337 independent reflections
4496 reflections with *I* > 2σ(*I*)
*R*_int_ = 0.033

#### Refinement

*R*[*F* ^2^ > 2σ(*F* ^2^)] = 0.035
*wR*(*F* ^2^) = 0.094
*S* = 1.00
5337 reflections
379 parameters
H-atom parameters constrained
Δρ_max_ = 0.22 e Å^−3^
Δρ_min_ = −0.21 e Å^−3^

### 

Data collection: *APEX2* (Bruker, 2007 ▶); cell refinement: *SAINT* (Bruker, 2007 ▶); data reduction: *SAINT*; program(s) used to solve structure: *SHELXTL* (Sheldrick, 2008 ▶); program(s) used to refine structure: *SHELXTL* and *FCF_filter* (Guzei, 2007 ▶); molecular graphics: *SHELXTL* and *DIAMOND* (Brandenburg, 1999 ▶); software used to prepare material for publication: *SHELXTL*, *publCIF* (Westrip, 2010 ▶) and *ModiCIFer* (Guzei, 2007 ▶).

## Supplementary Material

Crystal structure: contains datablock(s) global, I. DOI: 10.1107/S1600536812032801/zq2175sup1.cif

Structure factors: contains datablock(s) I. DOI: 10.1107/S1600536812032801/zq2175Isup2.hkl

Supplementary material file. DOI: 10.1107/S1600536812032801/zq2175Isup3.cml

Additional supplementary materials:  crystallographic information; 3D view; checkCIF report

## Figures and Tables

**Fig. 1.**
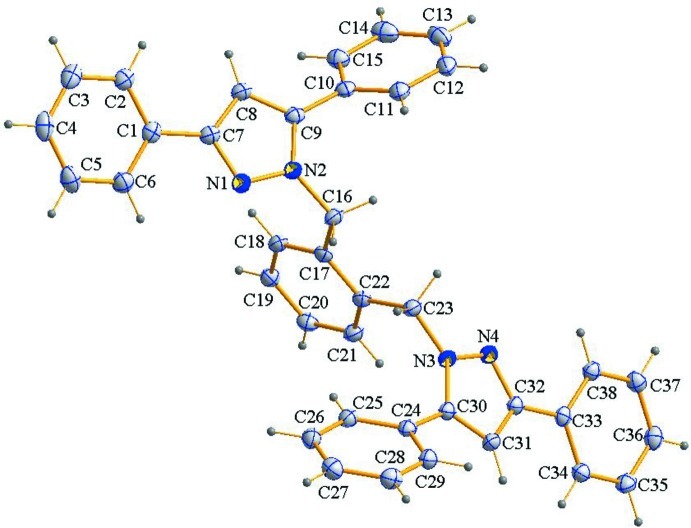
Molecular structure of (I) (Brandenburg, 1999). The thermal ellipsoids are shown at 50% probability level.

## supplementary crystallographic information

### Comment

Poly(pyrazol-1-ylmethyl)benzenes were first reported by Hartshorn & Steel (1995) and have subsequently been used to prepare metal complexes with interesting coordination structures (Hartshorn & Steel, 1997; Hartshorn & Steel, 1998; Motsoane *et al.*, 2007). The reactivity of these ligands depends on the steric size of substituents on the pyrazolyl ring. The phenyl substituents on the pyrazole rings of 1,2-bis(3,5-diphenylpyrazol -1-ylmethyl)benzene, compound (I), render the ligand sterically demanding and make it unable to ligate palladium complexes similarly to palladium complexes reported for less sterically crowded ligands where two ligands each bind to palladium in a monodentate fashion in a *trans* configuration (Motsoane *et al.*, 2007).

The bond distances and angles in (I) are unremarkable as confirmed by a *Mogul* structural check (Bruno *et al.*, 2002). The least squares planes defined by the pyrazole rings form a 21.09 (5)° angle between them. The lone pairs of electrons on N1 and N4 have a *trans*-like geometry and point in opposite directions of the disubstituted benzene ring with the N1—N2—N3—N4 torsion angle spanning 156.34 (10)°. The least squares planes of the two pyrazole rings form angles of 77.12 (4)° and 85.77 (4)° to the least squares plane of the central benzene ring (C17—C22). These angles and *trans*-like geometry are similar to those for the related compounds 1,2-bis((3-(2,2'-bipyridin-6-yl)pyrazol-1-yl)methyl)benzene with angles of 22.53°, 83.66°, and 85.34° and 2,3-bis((3-(2-pyridyl)pyrazol-1-yl)methyl)naphthalene with angles of 24.91°, 82.56°, and 82.56° (Al-Rasbi *et al.*, 2007; Paul *et al.*, 2003). The planes of the phenyl rings C1—C6 and C10—C15 form angles of 15.96 (6)° and 48.48 (4)° with the plane of the N1 pyrazole ring, and the planes of phenyl rings C24—C29 and C33—C38 form angles of 17.62 (6)° and 44.13 (3)° with the plane of the N3 pyrazole ring.

### Experimental

To a mixture of 1,2-bis(bromomethyl)benzene (1.50 g; 3.78 mmol) and 3,5-diphenylpyrazole (0.83 g; 3.78 mmol) in benzene (40 ml) was added 40% aqueous NaOH (12 ml) and 40% aqueous tetrabutylammonium bromide (10 drops). The mixture was then refluxed for 18 h. The crude product was washed with water (3 × 30 ml). The organic layer was separated, dried over anhydrous MgSO_4_ and evaporated *in vacuo* to afford the product as a white solid. Yield = 1.80 g (88%). ^1^H NMR (CDCl_3_): d7.38 (m, 20H,Ph-pz); 7.14 (dd, 2H, *^3^J* = 3.33 Hz, *^4^J* = 1.8 Hz), 6.90 (dd, 2H, *^3^J* = 3.33 Hz, *^4^J* = 1.8 Hz), 6.65 (s, 2H, H_4_-pz).

### Refinement

All H-atoms were placed in idealized locations and refined as riding with appropriate thermal displacement coefficients *U*_ĩso_(H) = 1.2 times *U*_eq_(bearing atom).

Default effective *X*—H distances for T = -173.0° C, C(*sp*^2^)–H=0.95, C(*sp*^3^)-2H=0.99.

### Crystal data

**Table:**

C_38_H_30_N_4_	*F*(000) = 1144
*M**_r_* = 542.66	*D*_x_ = 1.287 Mg m^−^^3^
Monoclinic, *P*2_1_/*c*	Cu *K*α radiation, λ = 1.54178 Å
Hall symbol: -P 2ybc	Cell parameters from 9916 reflections
*a* = 14.5338 (2) Å	θ = 3.2–71.0°
*b* = 13.6779 (2) Å	µ = 0.59 mm^−^^1^
*c* = 15.0051 (2) Å	*T* = 100 K
β = 110.102 (1)°	Block, colourless
*V* = 2801.18 (7) Å^3^	0.25 × 0.18 × 0.15 mm
*Z* = 4	

### Data collection

**Table:**

Bruker APEXII CCD diffractometer	5337 independent reflections
Radiation source: fine-focus sealed tube	4496 reflections with *I* > 2σ(*I*)
Graphite monochromator	*R*_int_ = 0.033
0.50° ω and 0.5 ° φ scans	θ_max_ = 71.8°, θ_min_ = 3.2°
Absorption correction: multi-scan (*SADABS*; Bruker, 2007)	*h* = −17→17
*T*_min_ = 0.891, *T*_max_ = 0.943	*k* = −15→13
44869 measured reflections	*l* = −18→17

### Refinement

**Table:**

Refinement on *F*^2^	Primary atom site location: structure-invariant direct methods
Least-squares matrix: full	Secondary atom site location: difference Fourier map
*R*[*F*^2^ > 2σ(*F*^2^)] = 0.035	Hydrogen site location: inferred from neighbouring sites
*wR*(*F*^2^) = 0.094	H-atom parameters constrained
*S* = 1.00	*w* = 1/[σ^2^(*F*_o_^2^) + (0.0515*P*)^2^ + 0.8417*P*] where *P* = (*F*_o_^2^ + 2*F*_c_^2^)/3
5337 reflections	(Δ/σ)_max_ = 0.001
379 parameters	Δρ_max_ = 0.22 e Å^−^^3^
0 restraints	Δρ_min_ = −0.21 e Å^−^^3^

### Special details

**Table:**

Geometry. All e.s.d.'s (except the e.s.d. in the dihedral angle between two l.s. planes) are estimated using the full covariance matrix. The cell e.s.d.'s are taken into account individually in the estimation of e.s.d.'s in distances, angles and torsion angles; correlations between e.s.d.'s in cell parameters are only used when they are defined by crystal symmetry. An approximate (isotropic) treatment of cell e.s.d.'s is used for estimating e.s.d.'s involving l.s. planes.
Refinement. Refinement of *F*^2^ against ALL reflections. The weighted *R*-factor *wR* and goodness of fit *S* are based on *F*^2^, conventional *R*-factors *R* are based on *F*, with *F* set to zero for negative *F*^2^. The threshold expression of *F*^2^ > σ(*F*^2^) is used only for calculating *R*-factors(gt) *etc*. and is not relevant to the choice of reflections for refinement. *R*-factors based on *F*^2^ are statistically about twice as large as those based on *F*, and *R*- factors based on ALL data will be even larger.

### Fractional atomic coordinates and isotropic or equivalent isotropic displacement parameters (Å^2^)

**Table:**

	*x*	*y*	*z*	*U*_iso_*/*U*_eq_	
N1	1.07757 (7)	0.35031 (8)	0.18839 (7)	0.0210 (2)	
N2	1.07756 (7)	0.25395 (8)	0.20801 (7)	0.0200 (2)	
N3	0.73795 (7)	0.03173 (8)	−0.00734 (7)	0.0195 (2)	
N4	0.74360 (7)	−0.06579 (8)	−0.01815 (7)	0.0206 (2)	
C1	1.19616 (9)	0.48327 (9)	0.23079 (9)	0.0208 (3)	
C2	1.28360 (9)	0.51980 (10)	0.29529 (9)	0.0234 (3)	
H2	1.3290	0.4763	0.3374	0.028*	
C3	1.30470 (10)	0.61878 (10)	0.29848 (10)	0.0283 (3)	
H3	1.3644	0.6426	0.3426	0.034*	
C4	1.23906 (11)	0.68315 (10)	0.23750 (10)	0.0306 (3)	
H4	1.2527	0.7512	0.2410	0.037*	
C5	1.15329 (10)	0.64754 (10)	0.17133 (10)	0.0302 (3)	
H5	1.1089	0.6913	0.1284	0.036*	
C6	1.13216 (10)	0.54873 (10)	0.16752 (10)	0.0263 (3)	
H6	1.0736	0.5250	0.1215	0.032*	
C7	1.17025 (9)	0.37968 (9)	0.23249 (8)	0.0198 (3)	
C8	1.22945 (9)	0.30099 (9)	0.27897 (9)	0.0209 (3)	
H8	1.2976	0.3027	0.3146	0.025*	
C9	1.16853 (9)	0.22083 (9)	0.26232 (8)	0.0195 (3)	
C10	1.19094 (9)	0.11972 (9)	0.29629 (8)	0.0218 (3)	
C11	1.13456 (10)	0.07016 (10)	0.34088 (9)	0.0264 (3)	
H11	1.0800	0.1018	0.3494	0.032*	
C12	1.15790 (11)	−0.02499 (11)	0.37277 (9)	0.0324 (3)	
H12	1.1185	−0.0588	0.4018	0.039*	
C13	1.23857 (12)	−0.07065 (11)	0.36219 (10)	0.0352 (4)	
H13	1.2540	−0.1361	0.3832	0.042*	
C14	1.29684 (11)	−0.02087 (11)	0.32089 (10)	0.0339 (3)	
H14	1.3532	−0.0517	0.3154	0.041*	
C15	1.27311 (10)	0.07339 (10)	0.28767 (9)	0.0265 (3)	
H15	1.3129	0.1068	0.2588	0.032*	
C16	0.98932 (9)	0.19824 (10)	0.15939 (9)	0.0217 (3)	
H16A	0.9316	0.2381	0.1568	0.026*	
H16B	0.9889	0.1389	0.1970	0.026*	
C17	0.97927 (8)	0.16768 (9)	0.05909 (8)	0.0187 (3)	
C18	1.04919 (9)	0.19193 (9)	0.01894 (9)	0.0206 (3)	
H18	1.1045	0.2298	0.0541	0.025*	
C19	1.03906 (9)	0.16123 (10)	−0.07241 (9)	0.0220 (3)	
H19	1.0873	0.1782	−0.0993	0.026*	
C20	0.95880 (9)	0.10598 (10)	−0.12399 (9)	0.0230 (3)	
H20	0.9516	0.0852	−0.1864	0.028*	
C21	0.88850 (9)	0.08090 (10)	−0.08410 (9)	0.0227 (3)	
H21	0.8335	0.0427	−0.1196	0.027*	
C22	0.89784 (8)	0.11112 (9)	0.00696 (8)	0.0187 (3)	
C23	0.82463 (9)	0.08275 (10)	0.05394 (8)	0.0208 (3)	
H23A	0.8581	0.0405	0.1092	0.025*	
H23B	0.8033	0.1427	0.0783	0.025*	
C24	0.61658 (9)	0.17018 (9)	−0.05221 (8)	0.0197 (3)	
C25	0.67272 (9)	0.24614 (10)	−0.06940 (8)	0.0215 (3)	
H25	0.7338	0.2319	−0.0766	0.026*	
C26	0.64017 (9)	0.34167 (10)	−0.07600 (9)	0.0241 (3)	
H26	0.6787	0.3925	−0.0884	0.029*	
C27	0.55158 (10)	0.36397 (10)	−0.06469 (9)	0.0256 (3)	
H27	0.5295	0.4298	−0.0689	0.031*	
C28	0.49552 (9)	0.28889 (10)	−0.04705 (9)	0.0254 (3)	
H28	0.4351	0.3036	−0.0387	0.030*	
C29	0.52705 (9)	0.19336 (10)	−0.04154 (9)	0.0224 (3)	
H29	0.4877	0.1427	−0.0304	0.027*	
C30	0.64550 (9)	0.06727 (9)	−0.05206 (8)	0.0190 (3)	
C31	0.58937 (9)	−0.01247 (9)	−0.09492 (8)	0.0203 (3)	
H31	0.5216	−0.0127	−0.1318	0.024*	
C32	0.65325 (9)	−0.09332 (9)	−0.07265 (8)	0.0191 (3)	
C33	0.63314 (9)	−0.19631 (9)	−0.10079 (9)	0.0194 (3)	
C34	0.54958 (9)	−0.22212 (9)	−0.17678 (9)	0.0216 (3)	
H34	0.5057	−0.1726	−0.2108	0.026*	
C35	0.52984 (10)	−0.31886 (10)	−0.20309 (9)	0.0249 (3)	
H35	0.4720	−0.3355	−0.2541	0.030*	
C36	0.59422 (10)	−0.39170 (10)	−0.15529 (9)	0.0247 (3)	
H36	0.5812	−0.4580	−0.1741	0.030*	
C37	0.67799 (9)	−0.36720 (10)	−0.07963 (9)	0.0240 (3)	
H37	0.7222	−0.4169	−0.0467	0.029*	
C38	0.69709 (9)	−0.27049 (10)	−0.05219 (9)	0.0214 (3)	
H38	0.7541	−0.2544	0.0000	0.026*	

### Atomic displacement parameters (Å^2^)

**Table:**

	*U*^11^	*U*^22^	*U*^33^	*U*^12^	*U*^13^	*U*^23^
N1	0.0204 (5)	0.0217 (6)	0.0207 (5)	−0.0014 (4)	0.0069 (4)	−0.0012 (4)
N2	0.0181 (5)	0.0224 (6)	0.0187 (5)	−0.0024 (4)	0.0053 (4)	−0.0012 (4)
N3	0.0158 (5)	0.0204 (6)	0.0216 (5)	−0.0019 (4)	0.0053 (4)	−0.0002 (4)
N4	0.0184 (5)	0.0201 (6)	0.0231 (5)	−0.0017 (4)	0.0067 (4)	0.0000 (4)
C1	0.0209 (6)	0.0227 (7)	0.0223 (6)	−0.0005 (5)	0.0121 (5)	−0.0029 (5)
C2	0.0226 (6)	0.0275 (8)	0.0218 (6)	−0.0020 (5)	0.0099 (5)	−0.0024 (5)
C3	0.0329 (7)	0.0309 (8)	0.0256 (7)	−0.0100 (6)	0.0157 (6)	−0.0079 (6)
C4	0.0437 (8)	0.0217 (8)	0.0353 (8)	−0.0055 (6)	0.0251 (7)	−0.0043 (6)
C5	0.0347 (8)	0.0246 (8)	0.0367 (8)	0.0049 (6)	0.0193 (6)	0.0054 (6)
C6	0.0236 (6)	0.0275 (8)	0.0288 (7)	0.0008 (5)	0.0102 (5)	0.0011 (5)
C7	0.0177 (6)	0.0243 (7)	0.0178 (6)	0.0000 (5)	0.0068 (5)	−0.0023 (5)
C8	0.0165 (6)	0.0255 (7)	0.0194 (6)	−0.0011 (5)	0.0044 (5)	−0.0017 (5)
C9	0.0174 (6)	0.0248 (7)	0.0164 (6)	−0.0005 (5)	0.0057 (5)	−0.0012 (5)
C10	0.0230 (6)	0.0226 (7)	0.0159 (6)	−0.0035 (5)	0.0016 (5)	−0.0028 (5)
C11	0.0268 (7)	0.0278 (8)	0.0210 (6)	−0.0064 (5)	0.0037 (5)	−0.0009 (5)
C12	0.0394 (8)	0.0288 (8)	0.0220 (7)	−0.0123 (6)	0.0016 (6)	0.0018 (5)
C13	0.0486 (9)	0.0211 (8)	0.0236 (7)	−0.0018 (6)	−0.0033 (6)	0.0001 (5)
C14	0.0356 (8)	0.0307 (9)	0.0283 (7)	0.0070 (6)	0.0018 (6)	−0.0042 (6)
C15	0.0260 (7)	0.0275 (8)	0.0236 (7)	0.0002 (5)	0.0052 (5)	−0.0022 (5)
C16	0.0161 (6)	0.0271 (7)	0.0205 (6)	−0.0050 (5)	0.0046 (5)	−0.0022 (5)
C17	0.0167 (6)	0.0181 (6)	0.0196 (6)	0.0021 (4)	0.0042 (5)	0.0020 (5)
C18	0.0154 (6)	0.0225 (7)	0.0219 (6)	−0.0009 (5)	0.0040 (5)	0.0001 (5)
C19	0.0184 (6)	0.0254 (7)	0.0234 (6)	0.0024 (5)	0.0089 (5)	0.0039 (5)
C20	0.0246 (6)	0.0256 (7)	0.0178 (6)	0.0023 (5)	0.0060 (5)	−0.0010 (5)
C21	0.0197 (6)	0.0238 (7)	0.0217 (6)	−0.0022 (5)	0.0035 (5)	−0.0022 (5)
C22	0.0157 (6)	0.0191 (7)	0.0198 (6)	0.0015 (4)	0.0041 (5)	0.0021 (5)
C23	0.0168 (6)	0.0238 (7)	0.0195 (6)	−0.0034 (5)	0.0034 (5)	−0.0012 (5)
C24	0.0189 (6)	0.0235 (7)	0.0150 (5)	−0.0023 (5)	0.0036 (5)	−0.0006 (5)
C25	0.0193 (6)	0.0243 (7)	0.0210 (6)	−0.0027 (5)	0.0070 (5)	−0.0011 (5)
C26	0.0270 (7)	0.0219 (7)	0.0237 (6)	−0.0047 (5)	0.0089 (5)	−0.0007 (5)
C27	0.0301 (7)	0.0231 (7)	0.0236 (6)	0.0023 (5)	0.0092 (5)	−0.0004 (5)
C28	0.0229 (6)	0.0309 (8)	0.0240 (6)	0.0024 (5)	0.0104 (5)	0.0010 (5)
C29	0.0207 (6)	0.0251 (7)	0.0215 (6)	−0.0021 (5)	0.0073 (5)	0.0020 (5)
C30	0.0163 (6)	0.0232 (7)	0.0179 (6)	−0.0016 (5)	0.0064 (5)	0.0023 (5)
C31	0.0156 (6)	0.0233 (7)	0.0206 (6)	−0.0021 (5)	0.0043 (5)	0.0019 (5)
C32	0.0165 (6)	0.0232 (7)	0.0182 (6)	−0.0020 (5)	0.0067 (5)	0.0018 (5)
C33	0.0183 (6)	0.0221 (7)	0.0206 (6)	−0.0014 (5)	0.0101 (5)	0.0015 (5)
C34	0.0205 (6)	0.0236 (7)	0.0202 (6)	0.0004 (5)	0.0063 (5)	0.0025 (5)
C35	0.0243 (6)	0.0285 (8)	0.0202 (6)	−0.0048 (5)	0.0057 (5)	−0.0023 (5)
C36	0.0300 (7)	0.0209 (7)	0.0257 (6)	−0.0031 (5)	0.0128 (5)	−0.0032 (5)
C37	0.0238 (6)	0.0230 (7)	0.0270 (7)	0.0031 (5)	0.0111 (5)	0.0031 (5)
C38	0.0175 (6)	0.0253 (7)	0.0219 (6)	−0.0015 (5)	0.0074 (5)	0.0010 (5)

### Geometric parameters (Å, º)

**Table:**

N1—C7	1.3420 (15)	C17—C22	1.4052 (17)
N1—N2	1.3505 (15)	C18—C19	1.3921 (18)
N2—C9	1.3700 (15)	C18—H18	0.9500
N2—C16	1.4541 (15)	C19—C20	1.3821 (18)
N3—N4	1.3495 (15)	C19—H19	0.9500
N3—C30	1.3683 (15)	C20—C21	1.3929 (18)
N3—C23	1.4571 (15)	C20—H20	0.9500
N4—C32	1.3405 (15)	C21—C22	1.3888 (17)
C1—C2	1.3986 (17)	C21—H21	0.9500
C1—C6	1.4004 (18)	C22—C23	1.5151 (16)
C1—C7	1.4686 (18)	C23—H23A	0.9900
C2—C3	1.385 (2)	C23—H23B	0.9900
C2—H2	0.9500	C24—C25	1.3988 (17)
C3—C4	1.386 (2)	C24—C29	1.4009 (17)
C3—H3	0.9500	C24—C30	1.4688 (18)
C4—C5	1.388 (2)	C25—C26	1.3815 (19)
C4—H4	0.9500	C25—H25	0.9500
C5—C6	1.383 (2)	C26—C27	1.3892 (19)
C5—H5	0.9500	C26—H26	0.9500
C6—H6	0.9500	C27—C28	1.3917 (19)
C7—C8	1.4045 (17)	C27—H27	0.9500
C8—C9	1.3770 (17)	C28—C29	1.3777 (19)
C8—H8	0.9500	C28—H28	0.9500
C9—C10	1.4713 (18)	C29—H29	0.9500
C10—C15	1.3967 (19)	C30—C31	1.3814 (17)
C10—C11	1.3984 (18)	C31—C32	1.4083 (17)
C11—C12	1.388 (2)	C31—H31	0.9500
C11—H11	0.9500	C32—C33	1.4708 (18)
C12—C13	1.385 (2)	C33—C34	1.3956 (17)
C12—H12	0.9500	C33—C38	1.3997 (17)
C13—C14	1.388 (2)	C34—C35	1.3825 (19)
C13—H13	0.9500	C34—H34	0.9500
C14—C15	1.383 (2)	C35—C36	1.3861 (19)
C14—H14	0.9500	C35—H35	0.9500
C15—H15	0.9500	C36—C37	1.3904 (18)
C16—C17	1.5200 (17)	C36—H36	0.9500
C16—H16A	0.9900	C37—C38	1.3848 (19)
C16—H16B	0.9900	C37—H37	0.9500
C17—C18	1.3880 (17)	C38—H38	0.9500
			
C7—N1—N2	105.07 (10)	C19—C18—H18	119.7
N1—N2—C9	112.33 (10)	C20—C19—C18	119.97 (11)
N1—N2—C16	117.92 (10)	C20—C19—H19	120.0
C9—N2—C16	129.02 (11)	C18—C19—H19	120.0
N4—N3—C30	112.48 (10)	C19—C20—C21	119.77 (11)
N4—N3—C23	118.30 (10)	C19—C20—H20	120.1
C30—N3—C23	129.01 (11)	C21—C20—H20	120.1
C32—N4—N3	105.29 (10)	C22—C21—C20	120.75 (12)
C2—C1—C6	118.33 (12)	C22—C21—H21	119.6
C2—C1—C7	120.67 (12)	C20—C21—H21	119.6
C6—C1—C7	120.93 (11)	C21—C22—C17	119.37 (11)
C3—C2—C1	120.72 (13)	C21—C22—C23	122.32 (11)
C3—C2—H2	119.6	C17—C22—C23	118.29 (10)
C1—C2—H2	119.6	N3—C23—C22	114.98 (10)
C2—C3—C4	120.26 (13)	N3—C23—H23A	108.5
C2—C3—H3	119.9	C22—C23—H23A	108.5
C4—C3—H3	119.9	N3—C23—H23B	108.5
C3—C4—C5	119.63 (13)	C22—C23—H23B	108.5
C3—C4—H4	120.2	H23A—C23—H23B	107.5
C5—C4—H4	120.2	C25—C24—C29	118.47 (12)
C6—C5—C4	120.31 (13)	C25—C24—C30	121.83 (11)
C6—C5—H5	119.8	C29—C24—C30	119.55 (11)
C4—C5—H5	119.8	C26—C25—C24	120.57 (12)
C5—C6—C1	120.70 (13)	C26—C25—H25	119.7
C5—C6—H6	119.7	C24—C25—H25	119.7
C1—C6—H6	119.7	C25—C26—C27	120.52 (12)
N1—C7—C8	110.81 (11)	C25—C26—H26	119.7
N1—C7—C1	120.01 (11)	C27—C26—H26	119.7
C8—C7—C1	129.12 (11)	C26—C27—C28	119.27 (13)
C9—C8—C7	105.91 (11)	C26—C27—H27	120.4
C9—C8—H8	127.0	C28—C27—H27	120.4
C7—C8—H8	127.0	C29—C28—C27	120.45 (12)
N2—C9—C8	105.88 (11)	C29—C28—H28	119.8
N2—C9—C10	124.78 (11)	C27—C28—H28	119.8
C8—C9—C10	129.32 (11)	C28—C29—C24	120.71 (12)
C15—C10—C11	119.00 (13)	C28—C29—H29	119.6
C15—C10—C9	119.26 (12)	C24—C29—H29	119.6
C11—C10—C9	121.68 (12)	N3—C30—C31	105.72 (11)
C12—C11—C10	120.37 (13)	N3—C30—C24	125.00 (11)
C12—C11—H11	119.8	C31—C30—C24	129.28 (11)
C10—C11—H11	119.8	C30—C31—C32	105.90 (10)
C11—C12—C13	119.97 (14)	C30—C31—H31	127.1
C11—C12—H12	120.0	C32—C31—H31	127.1
C13—C12—H12	120.0	N4—C32—C31	110.60 (11)
C12—C13—C14	120.04 (14)	N4—C32—C33	120.04 (11)
C12—C13—H13	120.0	C31—C32—C33	129.36 (11)
C14—C13—H13	120.0	C34—C33—C38	118.54 (12)
C15—C14—C13	120.25 (14)	C34—C33—C32	120.53 (11)
C15—C14—H14	119.9	C38—C33—C32	120.92 (11)
C13—C14—H14	119.9	C35—C34—C33	120.82 (12)
C14—C15—C10	120.32 (13)	C35—C34—H34	119.6
C14—C15—H15	119.8	C33—C34—H34	119.6
C10—C15—H15	119.8	C34—C35—C36	120.22 (12)
N2—C16—C17	114.13 (10)	C34—C35—H35	119.9
N2—C16—H16A	108.7	C36—C35—H35	119.9
C17—C16—H16A	108.7	C35—C36—C37	119.68 (12)
N2—C16—H16B	108.7	C35—C36—H36	120.2
C17—C16—H16B	108.7	C37—C36—H36	120.2
H16A—C16—H16B	107.6	C38—C37—C36	120.18 (12)
C18—C17—C22	119.48 (11)	C38—C37—H37	119.9
C18—C17—C16	121.91 (11)	C36—C37—H37	119.9
C22—C17—C16	118.59 (10)	C37—C38—C33	120.54 (12)
C17—C18—C19	120.65 (11)	C37—C38—H38	119.7
C17—C18—H18	119.7	C33—C38—H38	119.7
			
C7—N1—N2—C9	−1.03 (13)	C19—C20—C21—C22	0.26 (19)
C7—N1—N2—C16	−172.16 (10)	C20—C21—C22—C17	0.05 (19)
C30—N3—N4—C32	1.25 (13)	C20—C21—C22—C23	−178.07 (12)
C23—N3—N4—C32	176.48 (10)	C18—C17—C22—C21	−0.34 (18)
C6—C1—C2—C3	2.09 (18)	C16—C17—C22—C21	−178.73 (11)
C7—C1—C2—C3	−175.13 (11)	C18—C17—C22—C23	177.85 (11)
C1—C2—C3—C4	0.03 (19)	C16—C17—C22—C23	−0.54 (17)
C2—C3—C4—C5	−1.84 (19)	N4—N3—C23—C22	83.93 (13)
C3—C4—C5—C6	1.5 (2)	C30—N3—C23—C22	−101.75 (14)
C4—C5—C6—C1	0.7 (2)	C21—C22—C23—N3	−7.11 (18)
C2—C1—C6—C5	−2.44 (18)	C17—C22—C23—N3	174.76 (11)
C7—C1—C6—C5	174.77 (12)	C29—C24—C25—C26	−0.24 (18)
N2—N1—C7—C8	0.96 (13)	C30—C24—C25—C26	175.24 (11)
N2—N1—C7—C1	−176.53 (10)	C24—C25—C26—C27	0.67 (19)
C2—C1—C7—N1	163.03 (11)	C25—C26—C27—C28	−0.32 (19)
C6—C1—C7—N1	−14.11 (17)	C26—C27—C28—C29	−0.48 (19)
C2—C1—C7—C8	−13.94 (19)	C27—C28—C29—C24	0.92 (19)
C6—C1—C7—C8	168.91 (12)	C25—C24—C29—C28	−0.55 (18)
N1—C7—C8—C9	−0.56 (14)	C30—C24—C29—C28	−176.14 (11)
C1—C7—C8—C9	176.64 (12)	N4—N3—C30—C31	−0.82 (13)
N1—N2—C9—C8	0.70 (13)	C23—N3—C30—C31	−175.41 (11)
C16—N2—C9—C8	170.59 (11)	N4—N3—C30—C24	179.45 (11)
N1—N2—C9—C10	178.87 (11)	C23—N3—C30—C24	4.86 (19)
C16—N2—C9—C10	−11.24 (19)	C25—C24—C30—N3	45.84 (17)
C7—C8—C9—N2	−0.08 (13)	C29—C24—C30—N3	−138.73 (12)
C7—C8—C9—C10	−178.13 (12)	C25—C24—C30—C31	−133.82 (13)
N2—C9—C10—C15	133.94 (13)	C29—C24—C30—C31	41.61 (18)
C8—C9—C10—C15	−48.33 (18)	N3—C30—C31—C32	0.06 (13)
N2—C9—C10—C11	−48.95 (17)	C24—C30—C31—C32	179.77 (12)
C8—C9—C10—C11	128.77 (14)	N3—N4—C32—C31	−1.19 (13)
C15—C10—C11—C12	−2.44 (18)	N3—N4—C32—C33	178.62 (10)
C9—C10—C11—C12	−179.55 (11)	C30—C31—C32—N4	0.72 (14)
C10—C11—C12—C13	1.29 (19)	C30—C31—C32—C33	−179.07 (12)
C11—C12—C13—C14	0.9 (2)	N4—C32—C33—C34	−162.24 (11)
C12—C13—C14—C15	−1.9 (2)	C31—C32—C33—C34	17.52 (19)
C13—C14—C15—C10	0.7 (2)	N4—C32—C33—C38	17.77 (17)
C11—C10—C15—C14	1.45 (19)	C31—C32—C33—C38	−162.47 (12)
C9—C10—C15—C14	178.63 (11)	C38—C33—C34—C35	0.53 (18)
N1—N2—C16—C17	80.61 (14)	C32—C33—C34—C35	−179.46 (11)
C9—N2—C16—C17	−88.81 (15)	C33—C34—C35—C36	−1.34 (19)
N2—C16—C17—C18	−0.12 (17)	C34—C35—C36—C37	1.10 (19)
N2—C16—C17—C22	178.22 (11)	C35—C36—C37—C38	−0.05 (19)
C22—C17—C18—C19	0.35 (19)	C36—C37—C38—C33	−0.76 (18)
C16—C17—C18—C19	178.68 (12)	C34—C33—C38—C37	0.52 (18)
C17—C18—C19—C20	−0.05 (19)	C32—C33—C38—C37	−179.49 (11)
C18—C19—C20—C21	−0.25 (19)		
